# A disruptive sequencer meets disruptive publishing

**DOI:** 10.12688/f1000research.7229.1

**Published:** 2015-10-15

**Authors:** Nick Loman, Sarah Goodwin, Hans Jansen, Matt Loose

**Affiliations:** 1 Institute of Microbiology and Infection, University of Birmingham, Birmingham, UK; 2 Cold Spring Harbor Laboratory, Cold Harbour, NY, USA; 3 ZF-screens B.V., Leiden, Netherlands; 4 University of Nottingham, Nottingham, UK

**Keywords:** nanopore, MinION, sequencing, Oxford nanopore technology, MinION access programme, MinION analysis consortium

## Abstract

Nanopore sequencing was recently made available to users in the form of the Oxford Nanopore MinION. Released to users through an early access programme, the MinION is made unique by its tiny form factor and ability to generate very long sequences from single DNA molecules. The platform is undergoing rapid evolution with three distinct nanopore types and five updates to library preparation chemistry in the last 18 months. To keep pace with the rapid evolution of this sequencing platform, and to provide a space where new analysis methods can be openly discussed, we present a new *F1000Research* channel devoted to updates to and analysis of nanopore sequence data.

Nanopores are tiny hollow proteins, typically embedded in a membrane. DNA and RNA may be sequenced by monitoring electrical current changes as molecules block the nanopore and disrupt the flow of ions. These ideas have been explored and tinkered with in academic labs over 25 years ^1^. Recently, even the possibility of protein detection and characterisation has been proposed ^2^. But until recently nanopore sequencing has not made it into the hands of users. The first promises of a genuine sequencing product came at the Advances in Genome Biology and Technology (AGBT) 2012 in the form of an announcement from Oxford Nanopore Technologies. Attendees and observers were blown away by what was on offer: 100 kilobase long reads sequenced directly from blood, delivered in real-time ^3^. Perhaps most tantalisingly, this would be packaged up in a convenient USB stick sized device called the MinION.

An agonising delay followed to get the product to market, with manufacturing difficulties and a tiff with previous investor Illumina being blamed. Yet in May 2014, the first nanopore sequencers reached the hands of testers as part of the MinION Access Programme (MAP), an early access scheme rather different from roll-outs of other technologies. To reach a wide audience, MAP took a democratic approach and gave instruments to hundreds of labs, catering to highly diverse research interests. For many, including us, the device worked out of the box and delivered data (albeit small amounts and initially at low accuracy) as early as June 2014 ^4^. Since then, for the MAP participants it has been a white-knuckle ride. We have had three “pore” changes (R6, which was only around for about a month, R7 and the current version R7.3 ^5^). This was coupled with six chemistry changes and a software update seemingly every few weeks. For some it has been too much change, but early access programmes are not for everyone.

This pace of change has also taxed the community’s ability to reflect updates to the technology via traditional scientific publishing routes. As a consequence, in order to stay current, significant numbers of nanopore publications have been made available as preprints, with MAP participants being particularly enthusiastic adopters of the bioRxiv preprint server ( http://www.biorxiv.org). Later on they have been accepted as formal peer-reviewed publications. bioRxiv is an excellent place to deposit manuscripts, establishing priority and soliciting early input into papers - potentially catching issues with analysis and fostering community discussion. But the site is mainly a simple document archiving service, with most discussion occurring via social networking sites like Twitter.

*F1000Research* seeks to fill that gap - providing an intriguing mash-up of Science 2.0 functionality including key features expected of traditional journals such as permanent Digital Object Identifiers and PubMed indexing. It also functions something akin to a preprint server - articles, including the MinION Analysis Consortium (MARC) paper, are posted *prior* to peer review. The community can post comments on the articles, and peer reviews come in as they are ready. It's a disruptive model and seems perfectly suited to nanopore.

*F1000Research* also provides “channels”, either themed topically, or even associated with events like conferences. This channel is devoted to nanopore analysis.

The first manuscript to be published in the nanopore channel is an extensive dataset of *Escherichia coli* K-12 whole genome sequencing and analysis, the fruits of almost a year of analysis by the MARC ^6^. The consortium sequenced the same strain stock across five different laboratories on two continents, according to a strictly followed protocol including DNA extraction, shearing and library preparation. In so doing they have provided a snapshot of the repeatability of the instrument performance in different laboratories and provided deep technical details into all aspects of the sequencing protocol. The result is a huge corpus of nanopore data which will be of great value to those wishing to analyse and model signal, or “squiggle”, level information from the platform. They show evidence that the platform behaves very consistently between different labs. With this version of the platform (R7.3 pore, version 5 chemistry) the yields of high quality data from the instrument are becoming impressive, with up to several hundreds of megabases per run. Using the nanopore-optimised MarginAlign software ^7^ they report impressive accuracy scores of 92–93% when considering the filtered, best quality two-direction “passing” data.

The MARC analysis is as close we have to a Hayne’s manual ( https://en.wikipedia.org/wiki/Haynes_Manual) for nanopore sequencing on the MinION. This is not the last word in nanopore analysis, the platform has already seen a new chemistry update, and this paper has focused on a single bacterial species. In time, we hope that the *F1000Research* nanopore analysis channel will continue to provide snapshots of the technology as it evolves, and that this manuscript will be joined by many others to provide a definitive collection. We look forward to receiving your manuscripts.

## References

[1] Bayley H: Nanopore sequencing: from imagination to reality. *Clin Chem.* 2015;61(1):25–31. 10.1373/clinchem.2014.223016 25477535 PMC4404466

[2] Nivala J Marks DB Akeson M: Unfoldase-mediated protein translocation through an α-hemolysin nanopore. *Nat Biotechnol.* 2013;31(3):247–250. 10.1038/nbt.2503 23376966 PMC3772521

[3] Loman Lab Blog Post: Oxford Nanopore Megaton Announcement: Why do you need a machine?2012 Reference Source

[4] Loman N Quick J Calus S: A *P. aeruginosa* serotype-defining single read from our first Oxford Nanopore run. *figshare.* 2014 10.6084/m9.figshare.1052996

[5] Quick J Quinlan AR Loman NJ: A reference bacterial genome dataset generated on the MinION™ portable single-molecule nanopore sequencer. *Gigascience.* 2014;3:22. 10.1186/2047-217X-3-22 25386338 PMC4226419

[6] Ip C Loose M Tyson JR: MinION Analysis and Reference Consortium: Phase 1 data release and analysis. *F1000Research.* 2015 10.12688/f1000research.7201.1 PMC4722697 26834992

[7] Jain M Fiddes IT Miga KH: Improved data analysis for the MinION nanopore sequencer. *Nat Methods.* 2015;12(4):351–6. 10.1038/nmeth.3290 25686389 PMC4907500

